# Translation and Validation of the Spanish Version of the Banff Patellofemoral Instability Instrument 2.0

**DOI:** 10.1177/23259671241298306

**Published:** 2024-12-13

**Authors:** Juan Pablo Martinez-Cano, Maura D. Iversen, Alejandro Gallego, Luis Alfonso Gallon, Marie Askenberger, Johan von Heideken

**Affiliations:** * Departamento de Ortopedia, Fundación Valle del Lili, Cali, Colombia; † Universidad Icesi, Cali, Colombia; ‡ Department of Women’s and Children’s Health, Karolinska Institutet, Stockholm, Sweden; § College of Health and Wellness, Johnson & Wales University, Providence, Rhode Island, USA; ‖ Division of Rheumatology, Immunology & Immunity, Section of Clinical Sciences, Brigham & Women’s Hospital, and Department of Medicine, Harvard Medical School, Boston, Massachusetts, USA; Investigation performed at Departamento de Ortopedia, Fundación Valle del Lili, Cali, Colombia

**Keywords:** child, adolescent, patellofemoral instability, patellar dislocation, patient-reported outcome measure, translation, validation

## Abstract

**Background:**

The Banff Patellofemoral Instability Instrument (BPII) 2.0 is a patient-reported outcome measure (PROM) tailored specifically for patellofemoral instability. The BPII 2.0 was developed in English and has been validated for adolescents and translated into several languages, but not into Spanish.

**Purpose/Hypothesis:**

This investigation involved translating the BPII 2.0 into Spanish and evaluating and validating its psychometric properties. It was hypothesized that there would be a moderate correlation between the Spanish BPII 2.0 and the Spanish version of the Kujala score.

**Study Design:**

Cohort study (diagnosis); Level of evidence, 3.

**Methods:**

The BPII 2.0 underwent forward and backward translations into Colombian Spanish according to the Consensus-Based Standards for the Selection of Health Measurement Instrument guidelines. Colombian patients aged 9 to 18 years who experienced knee symptoms after a primary or recurrent patellar dislocation were recruited from a hospital-based orthopaedic clinic. Participants completed the Spanish BPII 2.0 and the Kujala score during their initial visit (t_0_) and the Spanish BPII 2.0 again 1 week later (t_1_). Internal consistency and test-retest reliability were assessed using the intraclass correlation coefficient (ICC). Concurrent validity of the Spanish BPII 2.0 with the Kujala score was explored through Pearson correlation analysis.

**Results:**

A total of 46 participants (31 [67%] female; mean age, 15.1 ± 2.0 years) were included. The mean time since first patellofemoral dislocation was 22 ± 28 months. Of the 4 participants who received operative treatment for patellar instability, the mean time since surgery was 12 months (range, 7-18 months). All patients completed the BPII 2.0 at t_0_ and at t_1_, a mean of 7 days later (range, 6-7 days), and 45 (98%) participants completed the Kujala score at t_0_. Five Spanish BPII 2.0 items exhibited floor or ceiling effects, however no subscales demonstrated these effects. Th Spanish BPII 2.0 demonstrated excellent internal consistency at both t_0_ (ICC, 0.94; 95% CI, 0.92-0.96) and t_1_ (ICC, 0.96; 95% CI, 0.93-0.97), along with excellent test-retest reliability (ICC, 0.98; 95% CI, 0.97-0.99). Concurrent validity of the Spanish BPII 2.0 with the Spanish Kujala score was good to strong (*r* = 0.74; 95% CI, 0.57-0.85).

**Conclusion:**

The Spanish BPII 2.0 had excellent internal consistency and test-retest reliability, suggesting this PROM is a reliable and valid questionnaire.

Among adolescents <15 years old with hemarthosis after acute knee injury who are evaluated at the emergency department, lateral patellar dislocation (LPD) is the most commonly reported diagnosis, with an incidence of 120 per 100,000 person-years.^3^ In the United States, the incidence has been reported as 148 per 100,000 person-years in adolescents aged 14 to 18 years,^38^ and in Colombia it has been reported as 188 per 100,000 person-years for the same age group.^27^ All 3 studies^3,27,38^ demonstrated that girls had a significantly greater incidence in the preadolescent population, while from the age of 14 years, LPD was more frequent in male patients.

LPD and recurrent instability may present significant challenges among children and adolescents, with ramifications including a compromised trust in knee function and knee pain and could present a considerable impact on quality of life.^32,33,40^ Furthermore, concerns about the potential associated cartilage damage heighten the risk of early onset of osteoarthritis.^31,41,45^ The recurrence rate after LPD is substantial, particularly in the presence of predisposing anatomical risk factors, with 30% to 70% experiencing a redislocation.^1,2,18,26,35^ Evaluating the outcomes of nonoperative and operative interventions requires the use of valid and reliable patient-reported outcome measures (PROMs) in conjunction with objective clinical assessments.^19^

Existing PROMs designed for patellofemoral instability include the Banff Patellofemoral Instability Instrument (BPII),^13^ its updated shorter version (BPII 2.0),^23^ and the Norwich Patellar Instability score.^42^ The BPII and the BPII 2.0 are quality-of-life outcome measures, and the Norwich Patellar Instability score is a knee symptom measure and does not currently exist in Spanish.^14^ Of these PROMs, only the BPII has undergone validation in an adolescent population with LPD and after patellofemoral stabilization.^24^ Endorsed by the International Patellofemoral Study Group, the BPII 2.0 has been translated from its original English into German,^4^ Dutch,^44^ Portuguese,^9^ Indonesian,^37^ Norwegian,^15^ and Swedish.^46^ However, there is a conspicuous absence of validated Spanish PROMs specifically tailored for the assessment of patellofemoral instability.

The Kujala anterior knee pain scale,^22^ also known as the Kujala score, is a PROM assessing subjective symptoms and functional limitations of patellofemoral dysfunction and anterior knee pain in adults and has recently been validated in adolescents.^17^ The English Kujala score has demonstrated moderate convergent validity with the English version of the BPII (*r* = 0.50; *P* < .001).^12^ The Kujala score has been translated into several languages, including Spanish.^10,25^ While the Kujala is a knee-specific questionnaire, only 1 item addresses patellar instability symptoms.^22^ Thus, its effectiveness in evaluating disabilities after LPD remains uncertain, potentially obscuring meaningful findings.

The primary objective of this study was to translate the BPII 2.0 into Spanish for use with children and adolescents with patellofemoral instability. Subsequently, we aimed to assess the concurrent validity of the Spanish version of the BPII 2.0 by comparing its scores with those of the Spanish version of the Kujala score.^25^ We hypothesized that a Spanish version of the BPII 2.0 would be valid and reliable in children and adolescents with patellofemoral instability.

## Methods

This cross-sectional study adhered to the Consensus-Based Standards for the Selection of Health Measurement Instruments (COSMIN) guidelines and framework for designing, evaluating, and reporting questionnaire properties after translation.^8,30^ This study, designed based on the Swedish translation of the BPII 2.0,^46^ involved selecting outcome measures that covered similar domains for participants in the same age range with the same diagnoses. A similar statistical methodology was used, and results were presented in a consistent manner. The research team has experience with translating and/or creating PROMs for adults and children.^7,11,20,25,28,34,46^ Ethical approval was obtained for the study protocol, and informed consent was obtained from all participants and caregivers before study involvement.

### Measures

The BPII 2.0, comprising 23 items, evaluates 5 domains crucial to quality of life. These domains encompass (1) symptoms and physical complaints (5 items), (2) work- and/or school-related concerns (4 items), (3) recreational/sport/activity (5 items), (4) lifestyle (5 items), and (5) social and emotional (4 items). Its validity, reliability, and responsiveness have been successfully demonstrated for assessing younger patients with lateral patellofemoral instability and following patellofemoral stabilization.^24^ Patient responses to items reflect their current knee status, function, and circumstances or beliefs related to the affected knee over the past 3 months. Patients record their current knee status by placing a mark on a 100-mm line, indicating a score between 0 and 100. A score of 0 represents greater symptoms and/or functional limitations and a lower quality of life, while a score of 100 indicates no symptoms and a higher quality of life. Each item carries equal weight, and the total score is calculated as the mean of all scores from answered items, ranging from 0 to 100.^23^

The Kujala score consists of 13 questions, with 13 items assessing lower extremity pain and physical alterations, 8 items evaluating limitations in function, and 2 items appraising the ability to participate in sports. Each question employs a Likert response set with 3 to 5 verbal anchors, and responses are rated from 0 to 10. The theoretical minimum score is 0, indicating severe symptoms, pain, and functional limitations, while the maximum possible score of 100 corresponds to an individual with healthy lower extremities, good physical condition, and no symptoms.^22^

### Translation of the BPII 2.0 Into Spanish

Forward and backward translations of the BPII 2.0 were performed according to international recommendations.^8,30^ An author (J.P.M.-C.) who was proficient in English and Spanish and who possessed extensive experience in patellar instability and dislocation in children, independently translated the BPII 2.0 from English to Colombian Spanish. A second bilingual individual, a paid professional translator who was an English and Spanish native speaker and a physician, independently translated the Spanish version of the BPII 2.0 back into English. Discrepancies between the backward translation and the original versions underwent review by the same paid translator, but no inconsistencies were identified. A team of researchers comprising orthopaedic surgeons with broad knowledge of patellofemoral instability examined the clarity and comprehensibility of the Spanish version, using a nominal group process to reconcile any differences. The final version of the Spanish BPII 2.0 was pilot tested among 5 patients aged 10 to 18 years. No changes were made after the piloting of the instrument, and the final version is available separately (see Supplemental Material).

### Recruitment of Participants

The sample size was determined following a precedent established by a study involving the translation of the BPII 2.0 into Swedish with 55 participants.^46^ In this convenience sample, potential participants with history of a primary or recurrent patellar dislocation, evaluated for patellofemoral instability following nonoperative or operative treatment, were identified at a single institution. All participants had a diagnosis of patellar dislocation with instability or recurrent patellar dislocation (International Classification of Diseases–10th Revision, codes S 83.0 or M 22.0^36^). The treating orthopaedic surgeon (J.P.M.-C.) confirmed the diagnosis based on the patient’s history, clinical examination, and imaging results (plain radiographs and magnetic resonance images). Demographic data were collected between July 2020 and June 2023 and included sex, date of birth, diagnosis, visit date, date of injury, side of injury (left, right, or bilateral) and date of operative or nonoperative treatment before survey administration.

Participants received an initial packet containing the Spanish BPII 2.0 and the Spanish Kujala score and completed these 2 questionnaires during their initial clinic visit. Subsequently, the participants were provided the Spanish BPII 2.0 questionnaire to complete at home 1 week after the initial visit, considering no interventions or changes in health status during this time frame. Participants were provided 3 options to return the completed survey: (1) take a photograph of the survey and send it by email or text message, (2) scan the form and email it back, or (3) mail the document by post to one of the authors (J.P.M.-C.), who recorded the data in a database. For surveys that were returned using a photo, the researcher measured the line and marking on the line and recorded the response based on the proportion of the line indicated.

### Statistical Analysis

Descriptive statistics were employed to characterize the cohort. The assessment of floor and ceiling effects utilized response frequency, with a threshold set at >15% of minimal-maximal values.^43^ Test-retest reliability, defined as the consistency of responses under repeated application of the measure and similar circumstances, was evaluated using intraclass correlation coefficients (ICCs) and 95% confidence intervals. Calculations were based on a 2-way, random, single-measure model with absolute agreement.

Internal consistency of the Spanish BPII 2.0 and the Spanish Kujala score was assessed using ICCs with 95% confidence intervals calculated based on a 2-way random, single-measure consistency model. While the Cronbach alpha is commonly employed for assessing internal consistency, the ICC was selected to allow for the computation of 95% confidence intervals.^5,16^ Interpretation criteria for internal consistency and test-retest reliability were as follows: ICCs <0.5 indicate poor reliability, between 0.5 and <0.74 are considered moderate, between 0.75 and 0.9 are good, and values >0.90 are indicative of excellent reliability.^21^

Concurrent validity of the Spanish BPII 2.0 with the Spanish Kujala score was assessed using the Pearson correlation coefficient, with 95% confidence intervals for these correlations estimated using the Fisher transformation method. Correlation coefficients (*r*s) in the ranges of 0.10-0.39 were deemed weak, 0.40-0.69 moderate, 0.70-0.89 good to strong, and 0.90-1.00 very strong.^39^ Given the conceptual similarity between the 2 measures, we hypothesized a moderate agreement between the Spanish BPII 2.0 and Spanish Kujala scores.

A post hoc sample size calculation was conducted using a power set at 80%, a significance level of .05 for a 1-sided test, and Pearson correlation of 0.74; the results indicated there was sufficient sample size.^6^

All statistical analyses were executed using IBM SPSS Statistics Version 29.

## Results

The study population consisted of 46 patients (31 female; 67.4%), with a primary (n = 18; 39%) or recurrent (n = 28; 61%) patellar dislocation evaluated for patellofemoral instability after nonoperative treatment (n = 42) or operative treatment (n = 4). A total of 21 participants had right knee involvement, 19 had left knee involvement, and 6 had bilateral involvement. The mean age of the patients was 15.1 ± 2.0 years (range, 9-18 years). The mean time since first dislocation was 22 ± 28 months (range, 0.5-120 months). Of the 4 participants who previously had operative treatment for patellofemoral instability, the mean time after surgery was 12 months (range, 7-18 months).

**Figure 1.**
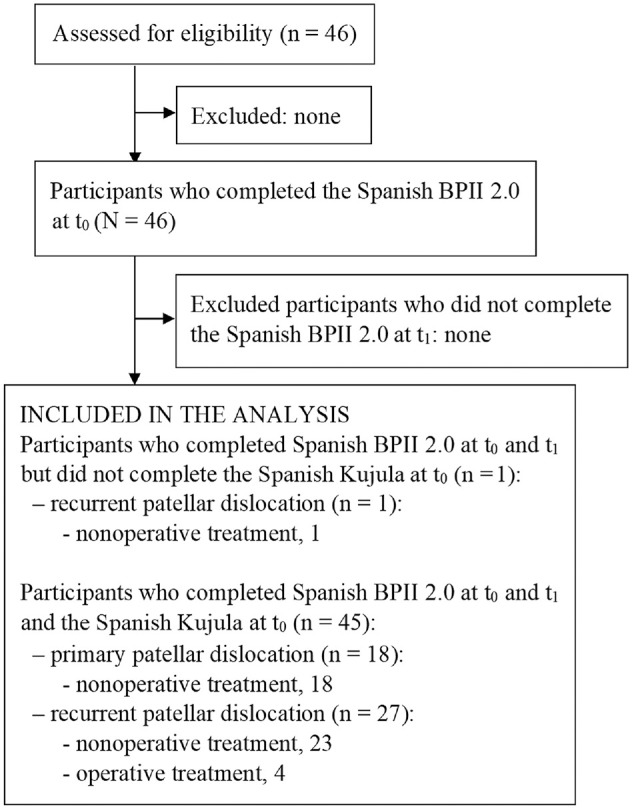
Flowchart of study participants with primary or recurrent patellar dislocation evaluated for patellofemoral instability after nonoperative or operative treatment. BPII, Banff Patellofemoral Instability Instrument.

All 46 participants answered every item on the questionannaires and completed the Spanish BPII 2.0 at baseline (t_0_) and at second administration (t_1_), at a mean of 7 days later (range, 6-7 days); and 45 participants (98%) completed the Spanish Kujala score at t_0_. Thus, analyses involving Spanish Kujala data or a combination of Spanish Kujala and BPII 2.0 data included only these 45 participants (1 participant with recurrent patellar dislocation who underwent nonoperative treatment was excluded). For analyses focusing on Spanish BPII 2.0 data, all 46 participants who responded to both questionnaires at t_0_ and t_1_ were included (Figure 1).

To assess the sensitivity and the breadth of items within the Spanish BPII 2.0, a threshold of >15% was used to identify ceiling and floor effects. On the individual item level, 5 items (items 1, 4, 7, 9, and 23) exceeded this threshold at t_0_ and 2 items (items 9 and 23) at t_1_. Item 1 demonstrated a floor effect, with 15% recording a zero for this item, indicating they were extremely troubled about their kneecap. Item 23 demonstrated a floor effect at both t_0_ and t_1_ with 20% and 22%, respectively, reporting they were extremely fearful of a reinjury. Items 4, 7, and 9 demonstrated ceiling effects (a score of 100). At t_0_, 17% of participants responded they had no loss of knee motion (item 4). For item 7, 16% reported no difficulty with squatting at t_0_. For item 9, a ceiling effect was found at both time points, with 20% and 22% of participants reporting no financial hardship due to their knee injury. Despite these 5 items demonstrating floor or ceiling effects, there were no floor or ceiling effects for BPII 2.0 subscales.

The mean Spanish BPII 2.0 scores at baseline and second administration were 49 (range 10-93) and 50 (range 4-89), respectively, suggesting little to no change in patient symptoms between the 2 survey administrations. The internal consistency of the total Spanish BPII 2.0 scores at t_0_ and t_1_ was excellent (ICC t_0_, 0.94 [95% CI, 0.92-0.96] and ICC t_1_, 0.96 [95% CI, 0.93-0.97]) with ICC values ranging from 0.62 to 0.89 for the different subscale scores across all administrations of the survey. The test-retest reliability of the Spanish BPII 2.0 total score was excellent, with an ICC of 0.98 (range, 0.97-0.99), and the ICCs for test-retest reliability of the subscales were also excellent (range, 0.90-0.97) (Table 1). The concurrent validity of the Spanish BPII 2.0 score with the Spanish Kujala score was good to strong (*r* = 0.74 [95% CI, 0.57-0.85]).

**Table 1:** Mean Scores, Internal Consistency, and Test-Retest Reliability of the Spanish BPII 2.0^*a*^

	Baseline (t_0_)		Follow-up (t_1_)		
	Mean (range)	Internal Consistency^*a*^ (95% CI)	Mean (range)	Internal Consistency^*a*^ (95% CI)	Test-Retest Reliability^*b*^ (95% CI)
Spanish BPII 2.0 score (N = 46 at t_0_ and t_1_)	49 (10-93)	0.94 (0.92-0.96)	50 (4-89)	0.96 (0.93-0.97)	0.98 (0.97-0.99)
Subscales					
Symptoms and physical complaints	57 (7-96)	0.74 (0.59-0.84)	55 (3-97)	0.79 (0.67-0.87)	0.90 (0.82-0.94)
Work- and/or school-related concerns	56 (4-100)	0.62 (0.40-0.77)	58 (5-100)	0.70 (0.53-0.82)	0.96 (0.93-0.98)
Recreation/sport/activity	38 (2-92)	0.84 (0.76-0.90)	40 (0-93)	0.88 (0.81-0.92)	0.94 (0.89-0.97)
Lifestyle	53 (7-100)	0.89 (0.83-0.93)	57 (5-98)	0.87 (0.80-0.92)	0.95 (0.90-0.98)
Social and emotional	41 (1-91)	0.82 (0.79-0.89)	40 (0-90)	0.84 (0.74-0.90)	0.97 (0.94-0.98)
Spanish Kujala score (n = 45)	72 (26-100)	0.81 (0.72-0.88)			

a Intraclass correlation coefficient, 2-way, random, consistency model.

b Intraclass correlation coefficient, 2-way, random, single measures with absolute agreement.

## Discussion

In this study, the BPII 2.0 was successfully translated into Colombian Spanish. This PROM is the first validated, disease-specific, and reliable questionnaire designed explicitly for patellofemoral instability in Spanish-speaking individuals. The test-retest reliability was excellent, with an ICC of 0.98 suggesting excellent reproducibility. This finding is in accordance with the validation of the original English BPII 2.0^23^ and the Swedish BPII 2.0,^46^ which both have an ICC of 0.97.

The mean Spanish BPII 2.0 total score was similar at t_0_ (49) in this cohort compared with previous translation studies evaluating recurrent patellar dislocation (range, 30-55),^4,9,44^ even though our sample included a large proportion of children and adolescents with patellofemoral instability who were treated nonoperatively (91.3%). The difference in mean Spanish BPII 2.0 scores between t_0_ and t_1_ was minimal and comparable with scores in the Swedish translation.^46^ The Spanish BPII 2.0 scores at t_0_ included a broad range of scores with values from 10 to 93, similar to previous translation studies of the BPII 2.0,^4,9,15,37,44,46^ indicating that patellofemoral instability represents a broad range of clinical symptoms affecting quality of life.

Floor and ceiling effects can affect the ability of a PROM to detect change because they limit the questionnaire’s ability to measure variance above or below a certain limit. A PROM with large floor or ceiling effects suggests that the measure may not be inclusive of all physical symptoms, functional limitations, or quality of life. Although some degree of floor and ceiling effects is expected, they make it difficult to distinguish among study participants at the top or bottom of a scale^29^ and may indicate the item is not challenging enough or may be too challenging. While there were 5 Spanish BPII 2.0 items (1, 4, 7, 9, 23) which demonstrated floor or ceiling effects, no ceiling or floor effects were identified for the Spanish BPII 2.0 subscales at each time point. These data are consistent with other translation studies of the BPII 2.0.^4,15,37,44,46^

The internal consistency (ICC) of the Spanish BPII 2.0 was excellent over both administrations (t_0_ = 0.94 and t_1_ = 0.96) and was comparable with the Cronbach alpha values reported in the original English version^23^ and other translations.^4,15,37,44,46^ These data suggest strong correlations among the items, with excellent reliability. There were no missing items in this study of the Spanish BPII 2.0, suggesting the wording was clear and patients did not find the questionnaire too long or repetitive.

The Spanish BPII 2.0 is a disease-specific questionnaire designed to evaluate patellofemoral instability, while the Kujala score is a questionnaire designed to evaluate a range of knee disorders, including patellar instability, and it contains only 1 item specific to patellofemoral instability. The correlation between total Spanish BPII 2.0 and Spanish Kujala was good to strong (*r* = 0.74), indicating that the Spanish Kujala score assesses different aspects of knee symptoms. In the German translation of the BPII 2.0,^4^ the authors assessed concurrent validity by comparing the German BPII 2.0 with the German versions of the Kujala score (*r*^2^ = 0.58) and Norwich Patellar Instability score (*r*^2^ = −0.47).

### Limitations and Strengths

This study has several potential limitations. First, the BP II 2.0 was translated into Colombian Spanish, so there is a possibility that individuals speaking different dialects of Spanish may interpret the items differently based on the wording. For example, *patella* is translated to *patella* in Colombian Spanish but is translated as *rótula* in Uruguayan Spanish. Although there are no wide variations in Spanish between different regions, minor differences may present a limitation. It is possible that the Colombian patients sampled for the initial comprehensibility study of the translated BPII 2.0 did not reveal cultural issues with the translation, even though none of the study participants indicated challenges with understanding the questions. With the participants completing the second administration of the translated BPII 2.0 at home, there was a potential for parental input on survey responses; however, if this were the case, one would expect greater differences in the mean and range of responses.

In addition, the study cohort included a majority of patients who had nonoperative treatment. Patients had their knees evaluated at a mean of 22 months, with a wide range (0.5-120 months), so the mean score on the Spanish BPII 2.0 may differ from that of other study samples. The evaluation of discrepancies between the backward translation and the original versions of the BPII 2.0 underwent review by the same paid translator and did not include the originator of the BPII 2.0 in this process. Response measurement (mark on 100-mm line) may have been affected by photographed survey responses from some patients, but this measurement error would be random. The sample size (N = 46) may have affected the robustness of the analysis; however, the post hoc sample size calculation indicated there was more than sufficient power for the study. Finally, the sample size could potentially affect the generalizability of these data to patients with different subjective symptoms of patellofemoral instability.

There are several strengths to this study. With approximately 500 million Spanish-speaking individuals in the world, a Spanish translation of the BPII 2.0 is an important contribution to the field. This research team has experience with both developing and translating PROMs and with the health condition under investigation. While a paid translator was hired for a component of the translation process, a 2-step process for review of the clarity and comprehensibility of the Spanish BPII 2.0 was conducted. Finally, this study followed the COSMIN recommendations for translation and testing,^8,30^ and 95% confidence intervals were provided for all statistical results to indicate the strength of the associations.

## Conclusion

The Spanish version of the BPII 2.0 demonstrated excellent internal consistency and test-retest reliability, indicating that it was a reliable and valid questionnaire to gather data on patellofemoral instability in Spanish-speaking patients. This new PROM can be used to complement clinical examinations and evaluate interventions for this population.

## Supplemental Material

sj-pdf-1-ojs-10.1177_23259671241298306 – Supplemental material for Translation and Validation of the Spanish Version of the Banff Patellofemoral Instability Instrument 2.0 Supplemental material, sj-pdf-1-ojs-10.1177_23259671241298306 for Translation and Validation of the Spanish Version of the Banff Patellofemoral Instability Instrument 2.0 by Juan Pablo Martinez-Cano, Maura D. Iversen, Alejandro Gallego, Luis Alfonso Gallon, Marie Askenberger and Johan von Heideken in Orthopaedic Journal of Sports Medicine
