# Supplementary material for: Translation and Validation of the Spanish Version of the Banff Patellofemoral Instability Instrument 2.0
Source: Orthop J Sports Med. 2024 Dec 13;12(12):23259671241298306. doi: 10.1177/23259671241298306 (PMC11645779; doi:10.1177/23259671241298306)
Supplement: sj-pdf-1-ojs-10.1177_23259671241298306 – Supplemental material for Translation and Validation of the Spanish Version of the Banff Patellofemoral Instability Instrument 2.0 [file sj-pdf-1-ojs-10.1177_23259671241298306.pdf]

Instrumento de BANFF para Inestabilidad Patelofemoral 2.0

Una Escala de Calidad de Vida para Pacientes con Inestabilidad Patelofemoral

Nombre del Paciente (nombre/apellido): ____________________________________

Fecha (día/mes/año): ____

Nombre de su cirujano:	Cuál rodilla le están viendo hoy?	Esta visita es su:
☐ ____________________	☐ Rodilla derecha<br>☐ Rodilla izquierda<br>☐ Ambas rodillas	☐ Primera consulta / examen<br>☐ Día de cirugía<br>☐ 3 meses postoperatorio<br>☐ 6 meses postoperatorio<br>☐ 12 meses postoperatorio<br>☐ 24 meses postoperatorio

INSTRUCCIONES

Por favor responda cada pregunta con respecto a su estado actual, función, circunstancias y creencias alrededor de la rodilla que tiene inestabilidad patelar. Considérelo para los últimos tres meses.

Indique con una barra oblicua (/) sobre la línea, el punto en el rango desde 0 hasta 100 que representa más cercanamente su situación.

Por ejemplo, la siguiente pregunta:

¿Este cuestionario es bueno?

0 ____ **100**

Inútil Fantástico

Si la barra oblicua fuera puesta en el centro de la línea, o en otras palabras, entre los extremos de 'inútil' y 'fantástico', esto indicaría que el cuestionario es considerado de calidad promedio. Es importante poner la barra oblicua al inicio o al final de la línea, si es que la descripción extrema refleja su situación.

Sección A: Síntomas y Molestias Físicas

1. ¿Qué tan preocupado/a lo tiene que su patela "salga de pronto" o sea inestable?

0 ______________________________ **100**

Extremadamente preocupado/a — No le preocupa en absoluto

2. ¿Qué tanto dolor o molestia siente en la rodilla con cualquier tipo de actividad prolongada (más de media hora)? Por ejemplo: estar de pie, caminar, hacer deporte, etc.

0 ______________________________ **100**

Dolor severo — No hay dolor en absoluto

3. ¿Qué tanto dolor o molestia siente en la rodilla al estar sentado/a mucho tiempo (más de media hora)? Por ejemplo: en cine, manejando, etc.

0 ______________________________ **100**

Dolor severo — No hay dolor en absoluto

4. ¿Tiene alguna pérdida de movilidad en su rodilla?

0 ______________________________ **100**

Pérdida severa de movilidad — No hay pérdida de movilidad

5. ¿Qué tan débil se siente su rodilla?

0 ______________________________ **100**

Extremadamente débil — No hay debilidad en absoluto

Sección B: Problemas Relacionados con el Trabajo y/o Escuela

**Si no está trabajando debido a su rodilla, marque la barra oblicua en el extremo izquierdo de cada línea.

6. ¿Cuánta dificultad tiene debido a su rodilla con los movimientos de giro o pivote en el trabajo y/o escuela?

0 ______________________________ **100**

Extrema dificultad — No hay dificultad en absoluto

7. ¿Cuánta dificultad tiene al hacer sentadillas en el trabajo y/o escuela?

0 ____ **100**

Extrema dificultad — No hay dificultad en absoluto

8. ¿Qué tanto le preocupa perder tiempo de trabajo y/o escuela debido al problema de su rodilla?

0 ____ **100**

Extrema preocupación — No le preocupa en absoluto

9. ¿Se han creado dificultades financieras para usted o su familia por la lesión en la rodilla?

0 ____ **100**

Severa dificultad financiera — No hay dificultad financiera

Sección C: Recreación / Deporte / Actividad

10. ¿Qué tan preocupado/a está porque sus actividades deportivas y/o recreativas puedan empeorar su rodilla?

0 ____ **100**

Extrema preocupación — No le preocupa en absoluto

11. ¿Al participar en actividad deportiva y/o recreativa debe hacerlo con precaución? (Marque con la barra oblicua el extremo de la izquierda, o sea 0, si es incapaz de participar en actividad recreativa o deportiva debido a su rodilla).

0 ____ **100**

Siempre con precaución — Nunca con precaución

12. Cuando participa en actividad deportiva y/o recreativa ¿Qué tanto temor tiene a que la rodilla "salga de pronto"? (Marque con la barra oblicua el extremo de la izquierda, o sea 0, si es incapaz de participar en actividad recreativa o deportiva debido a su rodilla).

0 ____ **100**

Temor extremo — No hay temor en absoluto

13. ¿Qué tanto le preocupa caminar sobre terreno irregular, una superficie húmeda o sobre hielo?

0 ____ **100**

Extrema preocupación — No le preocupa en absoluto

14. ¿Es capaz de dar su máximo esfuerzo en sus actividades deportivas y/o recreativas? (Marque con la barra oblicua el extremo de la izquierda, o sea 0, si es incapaz de participar en actividad recreativa o deportiva debido a su rodilla).

0 ____ **100**

Nunca es capaz — Siempre es capaz

Sección D: Estilo de Vida

15. ¿Qué tanto le preocupan sus cuestiones generales de seguridad debido al problema de su rodilla? Por ejemplo: subir o bajar por las escaleras, manejar, cargar niños pequeños, etc.

0 ____ **100**

Extrema preocupación — No le preocupa en absoluto

16. ¿Qué tanto se ha limitado su capacidad para ejercitarse y mantener la forma física debido al problema de su rodilla?

0 ____ **100**

Totalmente limitada — No se ha limitado en absoluto

17. ¿Qué tanto se ha limitado para disfrutar la vida debido al problema de su rodilla?

0 ____ **100**

Totalmente limitado/a — No se ha limitado en absoluto

18. ¿Evita actividades de estilo de vida con familia y/o amigos debido al problema de su rodilla?

0 ____ **100**

Siempre lo evita — Nunca lo evita

19. ¿Debe planear más que su familia y/o amigos las actividades de estilo de vida y sociales debido al problema de su rodilla?

0 ____ **100**

Siempre debe planear — Nunca debe planear

Sección E: Social y Emocional

20. ¿Le frustra que sus necesidades recreativas o competitivas ya no se cumplan debido al problema de su rodilla? (Marque con la barra oblicua el extremo de la derecha, o sea 100, si sus necesidad competitivas se cumplen. Marque con la barra oblicua el extremo de la izquierda, o sea 0, si no tiene necesidades competitivas).

0 ____ **100**

Extremadamente frustrado/a — No le frustra en absoluto

21. ¿Ha tenido dificultad para lidiar emocionalmente con el problema de su rodilla?

0 ____ **100**

Extrema dificultad — No se dificulta en absoluto

22. ¿Qué tan a menudo está nervioso/a por su rodilla?

0 ____ **100**

Siempre está nervioso/a — Nunca está nervioso/a

23. ¿Qué tan temeroso/a está de volver a lesionar su rodilla?

0 ____ **100**

Extremadamente temeroso/a — No está temeroso/a en absoluto

Gracias por responder este cuestionario.
